# Challenges in Rare Diseases Diagnostics: Incontinentia Pigmenti with Heterozygous *GBA* Mutation

**DOI:** 10.3390/diagnostics12071711

**Published:** 2022-07-14

**Authors:** Snežana Minić, Dušan Trpinac, Ivana Novaković, Nataša Cerovac, Danijela Dobrosavljević Vukojević, Jérémie Rosain

**Affiliations:** 1 A Clinics of Dermatovenerology, University Clinical Center of Serbia, Deligradska 34, 11000 Belgrade, Serbia; danijela_dobrosavljevic@yahoo.co.uk; 2 Faculty of Medicine, University of Belgrade, 11000 Belgrade, Serbia; natasa.cerovac.npk@gmail.com; 3 Institute of Histology and Embryology, Faculty of Medicine, University of Belgrade, Višegradska 26, 11000 Belgrade, Serbia; dusantrpinac@gmail.com; 4 Institute of Human Genetics, Faculty of Medicine, University of Belgrade, Višegradska 26, 11000 Belgrade, Serbia; tetaana61@yahoo.com; 5 Clinic for Neurology and Psychiatry for Children and Youth, University Clinical Center of Serbia, Dr. Subotica 6a, 11000 Belgrade, Serbia; 6 Laboratory of Human Genetics of Infectious Diseases, Necker Branch, INSERM U1163, Necker Hospital for Sick Children, 75015 Paris, France; jeremie.rosain@institutimagine.org; 7 Imagine Institute, University of Paris, 75015 Paris, France

**Keywords:** Incontinentia pigmenti, Gaucher disease, *IKBKG* gene mutations, *GBA* gene mutations, rare disease diagnostics, neurodegeneration, IFN-α2a, IFN-ω

## Abstract

Rare diseases represent a diagnostic challenge due to their number, variety of clinical phenomena, and possibility of a simultaneous presence of two or more diseases. An illustration of this challenge is an occurrence of a late diagnosis of a proband initially diagnosed with West syndrome, later revealed to be caused by Incontinentia pigmenti (IP). Furthermore, 20 years later, it was discovered that the proband was also a carrier of a heterozygous *GBA* gene mutation. The methods used in diagnostics were as follows: *IKBKG* gene analysis, the X-chromosome inactivation assay, analyses of the genes relevant for neurodegeneration, WES analysis, analysis of biochemical parameters typical for Gaucher disease (GD), and autoantibodies including IFN-α2a and IFN-ω. To avoid overlooking IP and other possible rare disease diagnoses, carefully searching for dermatological signs in these conditions is recommended. It is important that the diagnostic criteria are based on quality and extensive data from multiple studies of each rare disease. Establishing precise diagnostic criteria for as many rare diseases as possible and establishing a publicly accessible database of rare diseases with a search possibility according to phenotypic abnormalities and genetic mutations would greatly facilitate and speed up the establishment of an accurate diagnosis.

## 1. Introduction

Each individual rare disease is rare, but when considered as a group, rare diseases are common with a total prevalence of approximately 6–8% [1]. It is estimated that 263–446 million persons are affected globally at every moment in time [2]. Of all rare diseases, 71.9% are genetic [2]. Rare diseases always represent a diagnostic challenge due to their significant number and the variety of clinical phenomena. There is also a possibility of a simultaneous presence of two or more diseases or medical conditions in a patient. That is why they are often diagnosed as late or remain undiagnosed.

Such an example of late diagnosis is presented in this paper. This diagnostic odyssey lasted for a long period of time, as the proband was initially diagnosed with West syndrome. It was later discovered that West syndrome was caused by Incontinentia pigmenti (IP) and that the proband was a carrier of *GBA (Glucosylceramidase Beta)* gene mutation related to Gaucher disease (GD).

West syndrome is a constellation of symptoms characterized by epileptic/infantile spasms, abnormal brain wave patterns called hypsarrhythmia, and developmental arrest or regression [3]. It has an estimated birth prevalence of 3.7/100.000 [4]. Any disorder that can lead to brain damage can be an underlying cause of West syndrome, from trauma to genetic diseases [3]. This condition is currently regarded as a spectrum of disorders [3]. Pavone et al. [3] extensively analyzed genes involved in West syndrome, particularly infantile spasms. They made a list of 37 most frequent genes associated with infantile spasms. Neither the *IKBKG (inhibitor of kappa B kinase gamma)* gene nor the *GBA* gene, the causative genes of IP and GD, respectively, are among them. 

IP (OMIM 308300, Bloch–Sulzberger syndrome, ORPHA 464) is a rare X-linked genetic disorder [5] with an estimated prevalence of 1.2/100,000 [4]. It appears almost exclusively in females and is usually lethal in males [6]. The underlying cause of IP is a mutation of the *IKBKG (inhibitor of kappa B kinase gamma)* gene localized on the X-chromosome locus Xq28, which is the only gene known to be associated with IP [6]. The most frequent *IKBKG* gene mutation is an intragenic deletion encompassing exons 4-10, which is present in 75.1% of IP cases [7]. The total number of *IKBKG* mutation types detected so far is 170 [8]. The *IKBKG* gene product activates the nuclear factor kappa B (NF-κB) transcription factor, which regulates the expression of hundreds of genes in almost all cells [9]. Clinical manifestations of IP occur as a consequence of enhanced apoptosis due to this mutation [10]. The major diagnostic criteria for IP represent characteristic skin changes, alongside dental, ocular, and central nervous system (CNS) anomalies, which are considered as minor criteria [11]. In IP, CNS anomalies are severe and frequent, and they are found in 30.44% of IP patients, most of them being seizures [12]. It is possible for patients with the same *IKBKG* mutation to express very different signs and symptoms [13].

GD (OMIM * 606463, ORPHA 355) is an autosomal recessive inborn error of metabolism (lysosomal storage disease) due to the toxic accumulation of glucocerebroside lipids in various organs [14]. The underlying causes of all forms of GD are the homozygous or compound heterozygous mutations in the *GBA* gene, resulting in a lysosomal deficiency of glucocerebrosidase activity with a broad spectrum of phenotypes [14]. These mutations can also contribute to neurodegeneration [15]. GD has an estimated prevalence of 1.3/100,000 [4]. Similarly to IP, it is possible for patients with the same mutation to have very different signs and symptoms, such as severe joint pains, intellectual disability, seizures, Parkinsonism, and osteoporosis [14]. On the other hand, it is also possible for patients with similar signs and symptoms to have very different genetic mutations [14]. Environmental factors, as well as an individual’s particular genetic makeup, most likely influence the phenotypic expression of GD [14].

The aim of this study was to present and analyze a proband with a complex phenotype of IP combined with a heterozygous *GBA* mutation and to discuss the possibilities for an easier and faster diagnosis of such rare disease patients.

## 2. Materials and Methods

### 2.1. Genetic Analyses of the IKBKG Gene

DNA extracted from a peripheral blood sample was used for the molecular genetic examination, and the genetic testing of the proband and the proband’s family started with the confirmation of the *IKBKG* 4-10 exon deletion using the improved PCR method [16,17]. For sizing the PCR products, GeneRuler 1kb DNA ladder (Thermo Scientific, Waltham, MA, USA, cat. No SM0311) was used. Further molecular genetic analysis was performed in order to elucidate the proband’s complex phenotype.

### 2.2. The X-Chromosome Inactivation

The X-chromosome inactivation pattern was studied by the examination of the methylation status of the AR locus, as described elsewhere [18,19]. After a genomic DNA restriction’s digestion with enzymes *HpaII* (Thermo Fisher Scientific, Waltham, MA, USA, cat. No ER0511) and *RsaI* (Thermo Fisher Scientific, USA, cat. No ER1121), a PCR amplification of the selected AR locus region was performed, and products were separated on the ABI 3500 Genetic Analyzer (Life Technologies, Carlsbad, CA, USA).

### 2.3. Analysis of the Genes Relevant for Neurodegeneration

Additionally, a “clinical exome” next-generation sequencing (NGS) was performed following the manufacturer’s instruction [20] on an Illumina MiSeq platform (Illumina, San Diego, CA, USA) using TruSight One Panel (Illumina, USA) that includes coding the regions of 4813 genes associated with clinically relevant phenotypes. Using Illumina’s Variant Studio v.3.0, a data analysis was performed according to the phenotypic characteristics of the patient. A virtual gene panel was then created, comprising 185 genes relevant for neurodegeneration (Supplementary Materials). Only the variants that passed quality filters and had a global frequency of <5% were considered. NGS analysis singled out a heterozygous variant c.1448T>G (p.L444R) in the *GBA* gene. This result was then confirmed by Sanger sequencing [21], which was performed using a BigDye™ Terminator v3.1 Cycle Sequencing Kit (Applied Biosystems™, Waltham, MA, USA, cat. No 4337456) and ABI 3500 Genetic Analyzer (Life Technologies, USA). 

### 2.4. Whole Exome Sequencing (WES) Analysis

As Illumina TruSight One Panel does not include all genes, WES was performed by 3billion, Inc. Seoul, Republic of Korea, to make sure that there were no additional clinically significant variants/genes missed by the panel. Genomic DNA was extracted from whole blood using QIAamp DNA Blood Mini Kit (QIAGEN, Germantown, MD, USA). Exome capture was performed using xGen Exome Research Panel v2 (Integrated DNA Technologies, Coralville, IA, USA), and sequencing was performed using Novaseq 6000 (Illumina, San Diego, CA, USA). Sequencing data were processed as previously described [22]. Variant interpretation, including variant annotation, filtering, and classification, was performed using EVIDENCE, a software developed by 3billion [22]. Variants were classified following the ACMG guideline [23], and symptom similarity scores were calculated based on the patient’s phenotype using a scoring system developed by 3billion [22]. Variants were manually curated by the 3billion’s medical team.

### 2.5. Analysis of Biochemical Parameters Typical for GD

β-glucocerebrosidase and acid sphingomyelinase activities and glucosylsphinogosine (lyso-GL1) plasma levels were determined at Medical Laboratory Archimed Life Science GmbH, Vienna, Austria. Plasma chitotriosidase was investigated at the University Clinical Center of Serbia, Medical Biochemistry Center, Belgrade, Serbia.

### 2.6. Analysis of Autoantibodies Interferon-α2a (IFN-α2a) and IFN-ω

Screening of neutralizing activity toward type I interferon was performed as previously described [24] at the Laboratory of Human Genetics of Infectious Diseases, Necker Hospital for Sick Children, Paris, France, and University of Paris, Imagine Institute, Paris, France. Briefly, HEK293Ts were transfected with a luciferase plasmid containing 5 ISRE (GGGAAAGTGAAACTA) motifs and 1 renilla plasmid (pRL-SV40). Plasma or serum diluted 1 to 10 were incubated for 16 h in the presence or not of IFN-α2a (#130-108-984, Miltenyi Biotec) or IFN-ω (#300-02BC, Peprotech, London, UK) at the final concentration of 10 ng/mL or 100 pg/mL or in the presence of 10 ng/mL of IFN-β (#300-02BC, Peprotech). Results were read on a Victor (PerkinElmer, Waltham, MA, USA) using dual-luciferase reagents (#E1980, Promega, Madison, WI, USA) following the manufacturer’s instructions #E1980, Promega (Madison, WI, USA) [25]. 

## 3. Results

The proband was born at term, by vaginal vertex delivery, as the first-born child from the mother’s first pregnancy. The mother suffered no complications during the pregnancy, and the proband’s 5 min Apgar score was 8. At birth, the proband had neonatal seizures and skin changes in form of various pigmentation dispersed over the trunk and extremities, which, at the time, went undetected as suggestive of IP.

In the early postnatal period, there were clear delays in motor and mental maturation of the proband, and organic amblyopia was diagnosed. During this time, infantile spasms occurred and were continuously observed, and electroencephalography (EEG) showed hypsarrhythmia. The diagnosis of West syndrome was made, and an antiepileptic treatment with vigabatrin was initiated successfully.

At the age of two, the proband started experiencing focal epileptic seizures, which were subsequently and durably stopped by antiepileptic therapy with levetiracetam. In the following years, neurological examination revealed microcephaly and poor visual tracking. Furthermore, the proband presented severe delay in psychomotor development and was diagnosed as a spastic quadriplegic type of cerebral palsy. The proband was unable to sit or walk on her own, and the control of the sphincters was not achieved. These functional limitations were mostly due to spasticity, contractures, and joint deformities, and intensive physical and speech rehabilitation was, thus, performed. At the age of 15, the proband started experiencing joint pains. Osteoporosis was diagnosed at the age of 16, and the proband presented low bone mineral density the following year, at the age of 17.

At the age of 20, the diagnosis of IP was made (Table 1). This late diagnosis occurred only after the proband’s younger sister was examined at the age of 9 concerning skin changes that were characteristic for IP. Skin changes were subsequently observed on the proband’s mother’s skin as well. Following the genetic examination, both the proband’s mother (age of 40) and younger sister have since been diagnosed with IP, presenting *IKBKG* 4-10 exon deletion. By completing the diagnostic procedures, it was discovered that all three meet the updated IP diagnostic criteria [11] (Table 2). The proband was then referred to a neurological hospital for a further evaluation of her neurological status, revision of diagnosis, and confirmation of IP. 

In spite of the rehabilitation, the proband retained severe developmental delay and intellectual disability; they were unable to sit or move without support and were capable of speaking only in two-word sentences. 

The coexistence of numerous disabilities of which several did not fit into the IP criteria inspired further genetic analyses, and a heterozygous *GBA* mutation was revealed. Carriers of heterozygosity for *GBA* could be expected to have Parkinsonism, but the proband has not developed any signs of this disease so far.

The proband’s mother and younger sister had typical skin changes for IP, and the proband’s sister had severe dental anomalies (hypodontia, peg-like teeth) as well. All three of them had a gothic palate. Besides the aforementioned skin and dental and oral findings, the proband presented several other additional criteria, including CNS and ocular anomalies. These anomalies are suggestive of IP when combined, but exist as individual diagnoses, and were observed as such up until this moment. The proband’s brother had no clinical signs or symptoms of IP and was tested negative for *IKBKG* mutation. The proband’s father is deceased and, therefore, cannot be examined. 

The severity of anomalies in the proband, together with the proband’s developmental delay, led to suspicion whether there was another disorder in addition to IP that contributed to such a severe clinical picture. The complexity of clinical findings induced further genetic analysis, which then revealed the heterozygosity of the *GBA* gene.

### 3.1. Imaging Findings

**Figure 1:**
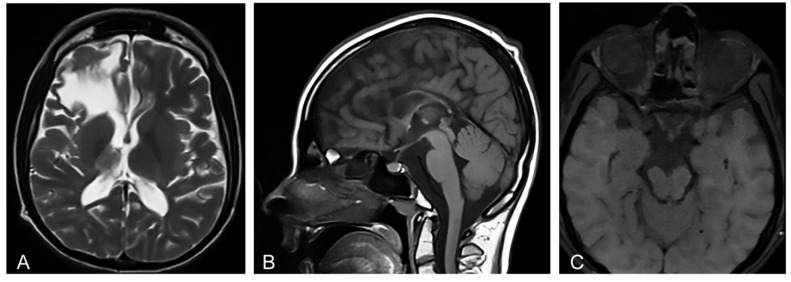
(**A**) Proband’s axial T2-weighted Magnetic resonance image (MRI) at the age of 20 shows abnormalities in supratentorial white matter and thalamus, and a porencephalic cyst in the right frontal lobe with the compression on the right lateral ventricle. (**B**) Proband’s sagittal T1-weighted MRI shows microcephaly and hypoplasia of the corpus callosum secondary to white matter loss. (**C**) Proband’s axial T1-weighted MRI shows an atrophy of the optic nerves, optic tracts, and optic chiasm.

Magnetic resonance imaging (MRI) of the brain showed microcephaly and supratentorial white matter abnormalities with cavity formation and gliotic alterations. In addition, MRI showed hypoplasia of corpus callosum and thalamus, as well as atrophy of truncus cerebri and the enlargement of the ventricular space (Figure 1A,B) that is characteristic for IP [12]. Orbital MRI showed an atrophy of the optic nerves, optic tracts, and optic chiasm (Figure 1C), corresponding to the findings of the concurrent ophthalmological examination. Transcranial ultrasonography of the brain showed normal echogenicity in the region of substantia nigra, and no pathological changes in the basal ganglia were observed at all. Ultrasonography of the abdomen did not show organomegaly characteristic of lysosomal storage disorders [14].

### 3.2. Genetic Findings

**Figure 2:**
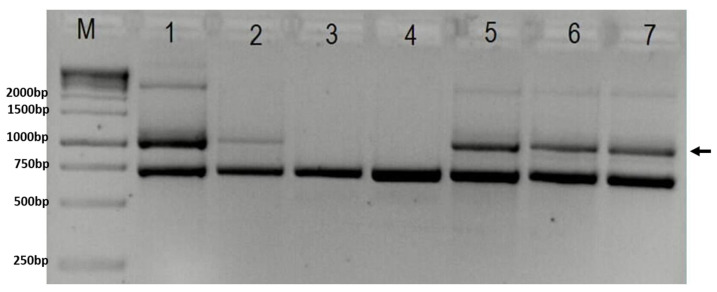
Gel-electrophoresis showing results of PCR test for deletion in the *IKBKG* gene (M—1 kB ladder; 1 and 2—positive controls; 3—negative control; 4—brother; 5—proband; 6—sister; 7—mother). Arrow indicates 1045 bp band present in case of deletion. The lower band represents 733 bp internal control. In column M is GeneRuler 1 kb DNA ladder (Thermo Scientific, USA, cat. No SM0311), with marked band size on the right.

**Figure 3:**
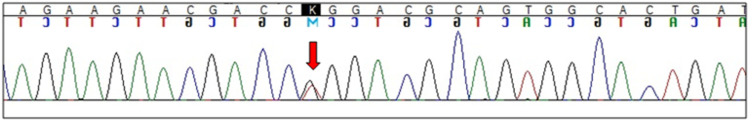
Electropherogram showing *GBA* L444R (c.1448T>G) mutation (arrow indicates heterozygous substitution).

Genetic analyses confirmed *IKBKG* 4-10 exon deletion (Figure 2). The X-chromosome inactivation analysis showed an apparently random pattern in the proband. Next Generation Sequencing (NGS) analysis singled out a heterozygous variant c.1448T>G (p.L444R) in the *GBA* gene (Figure 3). Exome sequencing performed at 3billion confirmed the heterozygous variant in *GBA* and did not identify any additional clinically significant variants. The presence of the heterozygous variant in *GBA* may have a certain impact on the proband’s phenotype (Table 3).

### 3.3. Analysis of Biochemical Parameters Typical for GD

β-glucocerebrosidase and acid sphingomyelinase activities (1.7 and 1.6 μmol/L/h, respectively) were above the cut-off value (>1.5 and >1.2, respectively). The quantitative measurement of lyso-GL1 was below (5.3 ng/mL) the cut-off value (0.0–14.0). Plasma chitotriosidase activity was (32.8 nmol/mL/h) in the range of reference values (1.80–146.6). β-glucocerebrosidase and acid sphingomyelinase activities were above the cut-off value. Quantitative measurement of lyso-GL1 was below the cut-off value.

### 3.4. Analysis of Autoantibodies IFN-α2a and IFN-ω

During the COVID-19 pandemic, it was suggested that the patients with the *IKBKG* mutation typical for IP (*IKBKG* 4–10 exon deletion) have a higher frequency of the presence of autoantibodies IFN-α2a and IFN-ω [26]. The proband and the proband’s sister tested positive for the presence of autoantibodies relative to both IFN-α2a and IFN-ω at high titer. The proband’s mother tested negative for the presence of these antibodies. None of the three IP carriers displayed neutralizing autoantibodies to type IFN-β.

## 4. Discussion

The initial diagnosis of the examined proband, West syndrome, is a diagnosis that includes a number of different diseases with similar symptoms. The subsequent evaluation of the proband’s diagnosis showed the presence of CNS anomalies as dominant, and a number of disorders of the skin, eyes, teeth, palate, and hair that belong to the diagnostic criteria of IP [6,11]. The diagnosis of IP was confirmed by genetic analysis and the detection of a mutation in the *IKBKG* gene. Due to the overlapping of similar findings and symptoms, there was a big delay in diagnosing the presented proband with IP and finally discovering the *GBA* mutation. Since the neurological changes were the most prominent ones of the different IP characteristics otherwise present in this proband, the proband’s diagnosis was, for a long period of time, reduced to the diagnosis of cerebral palsy, along with other neurological conditions. Cerebral palsy is a condition caused by multiple etiological factors and genetic and non-genetic factors such as teratogenic exposures, hypoxia, hemorrhage, or infections [27], which lead to neurological changes similar to those found in this proband.

In IP, both skewed and random X-chromosome inactivation were found [28]. In a group of IP patients, Dangouloff-Ros et al. [28] found severe neuroimaging anomalies associated with a random X-chromosome inactivation. The authors suggest that a skewed X-chromosome inactivation may protect the brain from damage, while, in the case of a random inactivation, the expression of the mutated *IKBKG* gene may lead to severe brain lesions. The findings of the examined proband’s random X-chromosome inactivation and severe brain anomalies are in accordance with this statement [28].

The proband’s complex symptomatology inspired the suspicion of some additional neurodegenerative diseases; therefore, the NGS analyses of 185 genes relevant for neurodegeneration and WES were performed. Besides the *IKBKG* gene mutation and a random X-chromosome inactivation, molecular genetic analysis in the proband showed heterozygous *GBA* gene mutation L444R, showing that the proband did not have GD, but it was a carrier for the *GBA* gene mutation. The proband’s phenotype is considered partially compatible with GD, which is an autosomal recessive disorder. However, because only a single heterozygous variant was detected, it is not yet possible to establish a molecular diagnosis. In the future, other genetic testing may be able to identify a second variant, such as deletion, copy number variation, duplication, and a deep intronic variant that was not detectable with the applied procedures.

The *GBA* gene encodes for the enzyme glucocerebrosidase. More than 500 different *GBA* mutations have been identified in patients affected by GD [29]. Biallelic *GBA* mutations are responsible for GD, while heterozygous *GBA* mutations are recognized as the most prominent genetic risk factor for idiopathic Parkinson’s disease [29,30,31,32]. However, no phenomenon of Parkinson disease was observed in the proband. The majority of GBA L444P heterozygotes in older adults cannot convert to PD [33]. In the Serbian population, the L444P mutations were not significantly associated with Parkinson’s disease, and there was no significant independent risk factor for Parkinson’s disease [21]. The role of *GBA* mutations in neurodegeneration is under extensive research. While the exact molecular mechanism is not yet understood, animal and cell culture studies are in favor of it, and they underline the role of metabolic disturbance and neuroinflammation [31,34]. The absence of Parkinsonism in the proband is consistent with previous findings [21] and the lack of MRI pathological findings in substantia nigra.

According to the phenotype displayed in the proband, it is unlikely that the concurrent *IKBKG* and heterozygous *GBA* gene mutations are coincidental. Besides the homozygous *GBA* mutation, a deficiency of glucocerebrosidase activity is expected for GD [14]. The presence of the heterozygous *GBA* mutation in the proband led to an examination of the biochemical parameters typical for GD [35] and, thus, the confirmation or exclusion of possible GD. In the proband, β-glucocerebrosidase and acid sphingomyelinase activities were above the cut-off value. The quantitative measurement of lyso-GL1 was below the cut-off value. Plasma chitotriosidase activity was within the reference values. All biochemical findings suggestive of GD for which analyses were performed were within the reference values, which indicates that GD is unlikely in the examined proband.

There are no data concerning the interrelation between the *IKBKG* gene product NF-κB and the *GBA* gene in the available literature [36]. The NF-κB proteins coordinate the expression of hundreds of genes [37] regulating key physiological processes such as inflammation, immunity, cell proliferation, and cell death [36]. Hypothetically, NF-κB may somehow have an influence on the *GBA* gene as well. The existence of the heterozygous variant in *GBA* may contribute to the explanation of the proband’s severe phenotype.

Microcephaly and the atrophy of corpus callosum and thalamus and of truncus cerebri are typical MRI CNS findings in IP [12], while the atrophy of the optic nerves, optic tracts, and optic chiasm was not reported in IP [12] nor in GD patients [38].

While the previously discussed skin, CNS, dental, and palate anomalies in the proband are typical for IP [5,12], severe joint pains and osteoporosis occur in GD. Some other typical signs and symptoms of GD such as hepatomegaly, splenomegaly, hypersplenism, pancytopenia, and Parkinsonism were not observed in the proband. If the proband was homozygous for *GBA* and consequently diagnosed with GD, these additional findings would be expected. Being heterozygous for *GBA*, according to available data, the presented proband would not be classified as GD but as a GD carrier and, therefore, should not have the clinical signs of GD. There is a possibility that the proband could have a second heterozygous mutation on *GBA* not detected by WES, such as deletion, copy number variation, duplication, and deep intronic variant. If that would be the case, the proband could be classified as GD.

The reported proband and the proband’s sister tested positive for the presence of autoantibodies relative to both IFN-α2a and IFN-ω at high titers. The proband’s mother tested negative for the presence of these antibodies. It has been shown that some women with IP have auto-Abs against type I IFNs [39]. Patients with positive autoantibodies relative to type I interferons are at a higher risk of severe COVID-19 disease [26].

The biggest challenge in making the correct rare disease diagnosis represents the limited experience of most physicians of different specializations. This is due to the low incidence of IP and other rare diseases, the existence of other syndromes with overlapping features/phenotypic abnormalities, and some mutations that may produce a spectrum of different anomalies. Therefore, by failing to consider that it is the combination of various conditions that makes for the diagnosis of a rare disease, this complex condition may be overlooked and treated solely as individual diagnoses that comprise it.

Another problem is the lack of precisely defined diagnostic criteria for all rare diseases. For example, in the presented proband, precise diagnostic criteria for IP have been established [6,11], and in the case of GD, five types of the disease have been identified, and a list of symptoms and signs has been created [14]. In both diseases, IP and GD, the genes on which a mutation leads to disease have been identified. It is important that the diagnostic criteria are based on quality and extensive data from multiple studies of each rare disease, as in the case of IP [11,12], and that experts who treat them participate in their definition. Therapeutic protocols for hereditary skin diseases are often allele-specific and require a thorough knowledge of genes and mutations. Confirming the diagnosis of the specific mutations can also be used for the identification, classification, and prognosis of the carriers, which are necessary for genetic counseling [40]. Establishing precise diagnostic criteria for as many rare diseases as possible and establishing an open access database of rare diseases with a search possibility according to phenotypic abnormalities and genetic mutations would provide a narrow list of possible rare diseases that can then be considered as diagnoses. This would greatly facilitate and speed up the establishment of an accurate diagnosis in the case of an rare undetected disease. To our knowledge, similar databases on rare diseases such as Orphanet [41,42] exist, but they exist on a small scale and without adequate search possibilities.

## 5. Conclusions

In order to avoid the overlooking of IP and other possible rare disease diagnoses, carefully searching for dermatological signs in these conditions is, therefore, recommended. In newborns without perinatal risk factors for cerebral palsy and other neurological disorders that present unexplained neurological deterioration or seizures, such as in the presented proband, searching for more precise explanations for the observed clinical findings is advised. Since neurological manifestations are prominent, polymorphic, and can occur at or soon after birth, while the initial skin lesions can be mild, this represents a particular challenge for the physicians in charge to understand the complexity of the patient’s condition. In conditions where mutations of certain known diseases are detected but clinical findings are beyond the diagnostic frames/criteria of such diagnosis, it would be advisable to perform further genetic analyses. Confirming the diagnosis in this manner leads to more accurate genetic counseling and helps in developing therapies for heritable skin diseases [40].

In the Orphanet database, the existence of more than 6000 different rare diseases annotated [4,42] makes it difficult to make an accurate diagnosis because no clinician is familiar with all of the rare diseases and the variants of their phenotypic abnormalities. Therefore, the existence of a publicly accessible database with phenotypic and genotypic abnormalities of rare diseases and the possibility of search according to these abnormalities would allow singling out possible rare diseases diagnoses. In that way, the physician can obtain a narrowed-down list of potential rare diseases of their patient, and they can quickly and easily achieve a more accurate diagnosis. The foundations for such a basis already exist but require significant expansion and improvements. The prevalence of all rare diseases of 6–8% [1] indicates that there is a need to intensify the work on making their diagnosis and to consider them as affecting patients more commonly than what is generally considered.

## Figures and Tables

**Table 1:** Basic data concerning the examined patients.

Patients	Proband	Sister	Mother
Age at moment of examination	20	10	40
Incontinentia pigmenti (IP) diagnosed according to the updated diagnostic criteria	+	+	+
Presence of *IKBKG* exons 4-10 deletion	+	+	+
Presence of heterozygous *GBA* mutation	+	−	−
Presence of some signs and symptoms of Gaucher disease (GD)	+	−	−
Presence of autoantibodies to both interferon-α2a (IFN-α2a) and IFN-ω	+	+	−
Vaccinated against COVID-19	−	−	−
Infected by COVID-19	−	−	−

**Table 2:** Presence of IP diagnostic criteria according to Minić et al. [11] in each patient.

IP Criteria/Patient	Proband	Sister	Mother
**Major criteria-stages**	3,4	3	3,4
**Minor criteria**			
Dental anomalies	+	+	+
Ocular anomalies	+	−	−
CNS (central nervous system) anomalies	+	−	−
Alopecia and abnormal hair	+	+	+
Abnormal nails	−	−	−
Palate anomalies	+	+	+
Nipple and breast anomalies	−	−	−
Multiple male miscarriages	−	−	−
Typical skin pathohistological findings	+	+	+
**Confirmed *IKBKG* gene mutation**	+	+	+

**Table 3:** The proband’s existing symptoms in comparison with the symptoms of Gaucher disease (GD) according to Stone et al. [14].

Symptoms and Signs of GD	Positive	Symptoms and Signs That May Also Be Considered as a Consequence of IP
Painless hepatomegaly and splenomegaly	−	−
Hypersplenism and pancytopenia	−	−
Severe joint pains, most frequently affecting hips and knees	+	−
Impaired olfaction and cognition (Type I)	+	Impaired cognition
Serious convulsions, hypertonia, intellectual disability, and apnea (Type II)	+	Serious convulsions, Hypertonia, intellectual disability
Myoclonus, seizures, dementia, and ocular muscle apraxia (Type III)	+	Myoclonus, seizures, dementia
Parkinsonism	−	−
Osteoporosis	+	−
Yellowish-brown skin pigmentation	+	Skin pigmentation

## Data Availability

The data presented in this study are available upon request from the corresponding author.

## Supplementary Materials

The following supporting information can be downloaded at: https://www.mdpi.com/article/10.3390/diagnostics12071711/s1, Virtual panel of 185 genes associated with neurodegeneration.
