# Supplementary material for: Challenges in Rare Diseases Diagnostics: Incontinentia Pigmenti with Heterozygous GBA Mutation
Source: Diagnostics (Basel). 2022 Jul 14;12(7):1711. doi: 10.3390/diagnostics12071711 (PMC9318020; doi:10.3390/diagnostics12071711)
Supplement: Supplementary file 1 [file diagnostics-12-01711-s001.zip › diagnostics-1790597-supplementary.pdf]

Supplementary material S1. Virtual panel of 185 genes associated with neurodegeneration.

ABCA7, ABCD1, ACE, ACOX1, ACMSD, ADAM10, APOC1, APOE, APOOP2, APP, ARSA, ARID5B, ASPA, ATP13A2, AUH, ATXN2, BCKDK, BIN1, BST1, C11orf30, CALHM1, CASS4, CCDC62, CD2AP, CD33, CELF1, CHCHD2, CHCHD10, CHMP2B, CHRNA9, CLU, CNTNAP2, COL4A2, COL4A1, COQ2, CR1, CRAT, CTC1, CSF1R, DARS2, DDRGK1, DGKQ, DLG2, DNAJC13, DNAJC5, DNAJC6, DNMT1, DSG2, DYRK1A, DYSF, ECHDC3, EIF2B, EIF4G1, EPHA1, ERCC6, ERCC8, EPHA4, FAM186A, FAM47E, FBF1, FBXO7, FGB, FGF20, FERMT2, FUS, GAB2, GAK , GALC, GBA, GBE1, GCDH, GCH1, GFAP, GLA, GPNMB, GRN, GSK3B, GUCY1A3, HEXA, HIP1R , HS3ST1, HTRA1, IL23R, INPP5D, INPP5F, ITGA8, ITPKB, KANSL1, KRT8P25, L2HGDH, LAMC2, LAMP3, LMNB1, LRRK2, LRRTM3, MADD, MAN2B1, MAPT, MC1R, MCCC1, MEF2C, MIR4697, MLC1, MMP16, MRPL38, MS4A4A, MTHFR, NCAM2, NEDD9, NLRP3, NME8, NMD3, NOTCH3, NSF, NUCKS1, NXPH1, NYAP1, OPTN, SERPINE1, PARK2, PARK7, PAXIP1, PICALM, PINK1, PLEKHM1, PLP1, PM20D1, PLD3, PRNP, PSAP, PSEN1, PSEN2, PTK2B, RAB7A, RAB7B, RAB25, RAB29, RAB38, RAI1, REST, RIN3, RIT2, RUNX1, SCARB2, SIPA1L2, SIRT2, SLC17A5, SLC2A13, SLC2A14, SLC41A1, SLC45A3, SLC7A4, SMPD1, SNCA, SORL1, SPPL2B, SQSTM1, SREBF1, STBD1, STK39, STX1B, SYT11, TARDBP, TBK1, TMEM106B, TMEM163, TMEM175, TMEM229B, TOMM40, TP63, TRIM47, TRIM65, TREM2, TREX1, TRIP4, TYMP, TYROBP, UNC5C, UPP2, USP25, VCP, VPS13C, VPS35, WBP2, ZCWPW1
